# Supplementary material for: Identification of novel biomarkers to distinguish clear cell and non-clear cell renal cell carcinoma using bioinformatics and machine learning
Source: PLoS One. 2024 Jun 10;19(6):e0305252. doi: 10.1371/journal.pone.0305252 (PMC11164351; doi:10.1371/journal.pone.0305252)
Supplement: S4 Table — (PDF) [file pone.0305252.s004.pdf]

**Supplemental table 4: The GO and pathway enrichment of specific DEGs of ccRCC**

| Category | Term | *p*-value | Gene counts |
|---|---|---|---|
| BP | GO:0006955~immune response | 8.71E-19 | 68 |
| BP | GO:0006954~inflammatory response | 1.86E-18 | 61 |
| BP | GO:0007166~cell surface receptor signaling pathway | 4.73E-14 | 47 |
| BP | GO:0042110~T cell activation | 1.23E-12 | 19 |
| BP | GO:0001525~angiogenesis | 3.19E-12 | 38 |
| BP | GO:0030199~collagen fibril organization | 7.51E-12 | 21 |
| BP | GO:0050852~T cell receptor signaling pathway | 1.29E-10 | 26 |
| BP | GO:0050870~positive regulation of T cell activation | 2.34E-10 | 14 |
| BP | GO:0007165~signal transduction | 4.16E-10 | 97 |
| BP | GO:0019882~antigen processing and presentation | 1.13E-09 | 15 |
| CC | GO:0005886~plasma membrane | 1.00E-26 | 348 |
| CC | GO:0009897~external side of plasma membrane | 2.85E-24 | 75 |
| CC | GO:0005887~integral component of plasma membrane | 2.60E-22 | 137 |
| CC | GO:0009986~cell surface | 2.55E-14 | 70 |
| CC | GO:0005576~extracellular region | 2.67E-14 | 155 |
| CC | GO:0016021~integral component of membrane | 8.81E-13 | 304 |
| CC | GO:0005615~extracellular space | 2.00E-11 | 137 |
| CC | GO:0042613~MHC class II protein complex | 2.58E-08 | 11 |
| CC | GO:0005581~collagen trimer | 1.01E-07 | 18 |
| CC | GO:0001772~immunological synapse | 1.13E-07 | 13 |

**Supplemental table 4: The GO and pathway enrichment of specific DEGs of ccRCC (Cont.)**

| Category | Term | *p*-value | Gene counts |
|---|---|---|---|
| MF | GO:0042802~identical protein binding | 1.67E-10 | 125 |
| MF | GO:0032395~MHC class II receptor activity | 8.65E-09 | 9 |
| MF | GO:0004888~transmembrane signaling receptor activity | 1.41E-08 | 28 |
| MF | GO:0005102~receptor binding | 5.08E-08 | 43 |
| MF | GO:0023026~MHC class II protein complex binding | 8.97E-08 | 11 |
| MF | GO:0030020~extracellular matrix structural constituent conferring tensile strength | 5.56E-07 | 12 |
| MF | GO:0038023~signaling receptor activity | 2.01E-06 | 27 |
| MF | GO:0005044~scavenger receptor activity | 5.75E-06 | 12 |
| MF | GO:0045028~G-protein coupled purinergic nucleotide receptor activity | 6.46E-06 | 7 |
| MF | GO:0005515~protein binding | 1.46E-05 | 575 |
