# Supplementary material for: Identification of novel biomarkers to distinguish clear cell and non-clear cell renal cell carcinoma using bioinformatics and machine learning
Source: PLoS One. 2024 Jun 10;19(6):e0305252. doi: 10.1371/journal.pone.0305252 (PMC11164351; doi:10.1371/journal.pone.0305252)
Supplement: S5 Table — (PDF) [file pone.0305252.s005.pdf]

Supplemental table 5: The GO and pathway enrichment of specific DEGs of pRCC

Category	Term	*p*-value	Gene counts
BP	GO:0051216~cartilage development	9.03E-05	8
BP	GO:0061436~establishment of skin barrier	1.57E-04	6
BP	GO:0003341~cilium movement	1.79E-04	7
BP	GO:0003351~epithelial cilium movement	3.55E-04	5
BP	GO:0036158~outer dynein arm assembly	8.64E-04	5
BP	GO:0016042~lipid catabolic process	0.001655	8
BP	GO:0044458~motile cilium assembly	0.001748	5
BP	GO:0010466~negative regulation of peptidase activity	0.001985	7
BP	GO:0070286~axonemal dynein complex assembly	0.002054	4
BP	GO:0098609~cell-cell adhesion	0.002165	11
CC	GO:0005615~extracellular space	4.99E-07	65
CC	GO:0005576~extracellular region	2.17E-06	67
CC	GO:0031594~neuromuscular junction	4.93E-04	8
CC	GO:0070062~extracellular exosome	6.45E-04	60
CC	GO:0005737~cytoplasm	7.29E-04	127
CC	GO:0005930~axoneme	0.001288	9
CC	GO:0005788~endoplasmic reticulum lumen	0.002934	14
CC	GO:0035580~specific granule lumen	0.0045	6
CC	GO:0071944~cell periphery	0.004819	6
CC	GO:0000785~chromatin	0.010543	30

Supplemental table 5: The GO and pathway enrichment of specific DEGs of pRCC (Cont.)

Category	Term	*p*-value	Gene counts
MF	GO:0005102~receptor binding	0.001097	18
MF	GO:0004867~serine-type endopeptidase inhibitor activity	0.003215	8
MF	GO:0008201~heparin binding	0.004235	10
MF	GO:0042802~identical protein binding	0.007465	45
MF	GO:0008009~chemokine activity	0.011979	5
MF	GO:0005198~structural molecule activity	0.023284	9
MF	GO:0045236~CXCR chemokine receptor binding	0.028522	3
MF	GO:0045504~dynein heavy chain binding	0.032219	3
MF	GO:0003990~acetylcholinesterase activity	0.03532	2
MF	GO:0005427~proton-dependent oligopeptide secondary active transmembrane transporter activity	0.03532	2
