# Supplementary material for: Identification of novel biomarkers to distinguish clear cell and non-clear cell renal cell carcinoma using bioinformatics and machine learning
Source: PLoS One. 2024 Jun 10;19(6):e0305252. doi: 10.1371/journal.pone.0305252 (PMC11164351; doi:10.1371/journal.pone.0305252)
Supplement: S6 Table — (PDF) [file pone.0305252.s006.pdf]

Supplemental table 6: The GO and pathway enrichment of specific DEGs of chRCC

Category	Term	*p*-value	Gene counts
BP	GO:1902600~hydrogen ion transmembrane transport	4.34E-08	20
BP	GO:0007035~vacuolar acidification	1.20E-05	8
BP	GO:0097401~synaptic vesicle lumen acidification	1.33E-05	7
BP	GO:0072659~protein localization to plasma membrane	2.38E-05	19
BP	GO:0051452~intracellular pH reduction	9.55E-05	6
BP	GO:0007595~lactation	9.70E-05	9
BP	GO:0006629~lipid metabolic process	1.28E-04	21
BP	GO:0042472~inner ear morphogenesis	1.73E-04	10
BP	GO:0008203~cholesterol metabolic process	2.03E-04	12
BP	GO:0006887~exocytosis	2.58E-04	14
CC	GO:0016021~integral component of membrane	5.94E-06	249
CC	GO:0005886~plasma membrane	3.02E-05	242
CC	GO:0005765~lysosomal membrane	5.31E-05	31
CC	GO:0033176~proton-transporting V-type ATPase complex	6.11E-05	6
CC	GO:0005764~lysosome	6.40E-05	27
CC	GO:0016020~membrane	6.93E-05	177
CC	GO:0005743~mitochondrial inner membrane	7.42E-05	36
CC	GO:0005739~mitochondrion	7.57E-05	81
CC	GO:0016323~basolateral plasma membrane	8.54E-05	23
CC	GO:0016471~vacuolar proton-transporting V-type ATPase complex	1.34E-04	6

Supplemental table 6: The GO and pathway enrichment of specific DEGs of chRCC (Cont.)

Category	Term	*p*-value	Gene counts
MF	GO:0003779~actin binding	2.14E-07	35
MF	GO:0046961~proton-transporting ATPase activity, rotational mechanism	2.59E-05	8
MF	GO:0030276~clathrin binding	2.27E-04	9
MF	GO:0030506~ankyrin binding	5.34E-04	6
MF	GO:0005516~calmodulin binding	6.91E-04	19
MF	GO:0015078~hydrogen ion transmembrane transporter activity	0.001447	7
MF	GO:0005096~GTPase activator activity	0.00146	23
MF	GO:0000146~microfilament motor activity	0.001967	7
MF	GO:0051015~actin filament binding	0.004283	18
MF	GO:0005546~phosphatidylinositol-4,5-bisphosphate binding	0.004351	10
